# Supplementary material for: Stability of person-specific blood-based infrared molecular fingerprints opens up prospects for health monitoring
Source: Nat Commun. 2021 Mar 8;12:1511. doi: 10.1038/s41467-021-21668-5 (PMC7940620; doi:10.1038/s41467-021-21668-5)
Supplement: Supplementary file 1 — Supplementary Information [file 41467_2021_21668_MOESM1_ESM.pdf]

Stability of person-specific blood-based infrared molecular fingerprints opens up prospects for health monitoring

SUPPLEMENTARY INFORMATION

Supplementary Table 1: *Breakdown of the cohort of study participants. Apart from the listed comorbidities, none of the participants had a known disease or showed any symptoms of any disease.*

ID	Sex	Age	BMI	Smoking Status	Comorbidities
AA	female	28	n/a	n/a	
AB	female	54	23.1	non-smoker	
AC	male	29	n/a	n/a	
AD	female	54	21.2	active-smoker	
AE	female	55	22.4	active-smoker	high blood pressure
AF	female	24	26.6	non-smoker	allergies
AG	female	23	n/a	n/a	
AH	female	31	18.4	non-smoker	
AI	female	45	22.4	non-smoker	allergies
AJ	female	39	n/a	non-smoker	
AK	female	24	n/a	n/a	
AL	male	55	21.8	non-smoker	
AM	male	20	20.6	non-smoker	allergies
AN	female	42	20.2	non-smoker	
AO	male	46	23.4	non-smoker	
AP	female	28	22.8	non-smoker	
AQ	male	39	25.7	non-smoker	
AR	male	28	22.2	non-smoker	
AT	male	32	n/a	non-smoker	
AU	male	58	28.6	non-smoker	
AV	female	26	19.5	non-smoker	fibroma
AW	male	61	n/a	non-smoker	
AX	male	32	25	non-smoker	
AY	male	56	24.1	active-smoker	psoriasis
AZ	male	32	n/a	non-smoker	
BB	female	27	n/a	n/a	
BC	male	70	n/a	non-smoker	
BD	female	19	18.2	non-smoker	
BE	female	38	38.8	non-smoker	
BF	male	53	31.8	ex-smoker	
BG	male	27	n/a	non-smoker	

Supplementary Table 2: *Overview of the sampling time of all collected blood samples. Since one serum and one plasma sample was collected per individual at a time, the absolute number of samples is 636. Depending on the number of blood samples per individual, the corresponding data were used for different types of evaluation. All data were taken into account when calculating the between- and within person variability of the infrared fingerprints and its associated spectral markers. Only data from individuals with more than 7 blood draws within the first 7 weeks were included in the training and test of the classification models for the identification of individuals (green shaded entries). The test of the long-term stability of infrared fingerprints was only performed on data from individuals that donated again 6 months after the first sampling period (dark green shaded areas).*

ID	Day(s) in the course of the study															Total number of blood draws	
	1	7	9	14	17	21	24	28	31	35	38	42	45	233	238	Day 1-45	Day 233-238
AA		x	x	x	x	x		x		x	x	x				9	0
AB				x	x	x	x	x	x	x	x	x	x	x	x	10	2
AC	x	x	x	x	x			x	x	x	x					9	0
AD	x	x	x		x	x	x	x					x	x	x	8	2
AE	x	x	x	x	x	x	x	x			x		x	x	x	10	2
AF	x	x	x	x	x	x		x	x	x	x		x	x		11	1
AG	x	x	x	x												4	0
AH	x	x	x			x	x	x	x	x	x	x	x	x	x	11	2
AI	x	x	x	x	x			x		x	x	x	x		x	10	1
AJ	x	x	x	x		x	x	x	x	x	x					10	0
AK	x	x			x	x		x	x	x	x	x				9	0
AL	x	x	x	x	x	x	x					x	x	x		9	1
AM	x	x	x	x	x	x	x	x	x	x				x	x	10	2
AN	x	x	x	x	x	x	x					x	x	x	x	9	2
AO	x			x	x	x	x	x	x	x	x	x		x	x	10	2
AP	x	x	x		x		x	x	x	x	x			x	x	9	2
AQ	x	x	x	x	x		x	x	x	x				x		9	1
AR	x	x	x	x	x	x								x	x	6	2
AT	x	x	x	x	x	x		x	x	x	x	x	x			12	0
AU	x	x	x	x	x		x	x	x	x				x	x	9	2
AV	x	x	x	x	x		x			x	x	x	x	x	x	10	2
AW	x	x		x	x	x	x	x	x	x						9	0
AX	x	x	x		x			x	x		x	x	x	x		9	1
AY	x	x	x	x	x	x	x	x			x		x	x	x	10	2
AZ	x	x	x	x	x	x	x	x	x		x	x				11	0
BB	x	x	x					x	x	x	x		x			8	0
BC		x		x	x	x	x	x	x	x	x		x			10	0
BD		x	x	x	x	x	x	x	x	x	x		x	x	x	11	2
BE			x	x	x	x	x	x	x	x	x	x	x	x		11	1
BF				x	x	x		x		x	x			x		6	1
BG						x	x	x	x		x		x			6	0
																318	

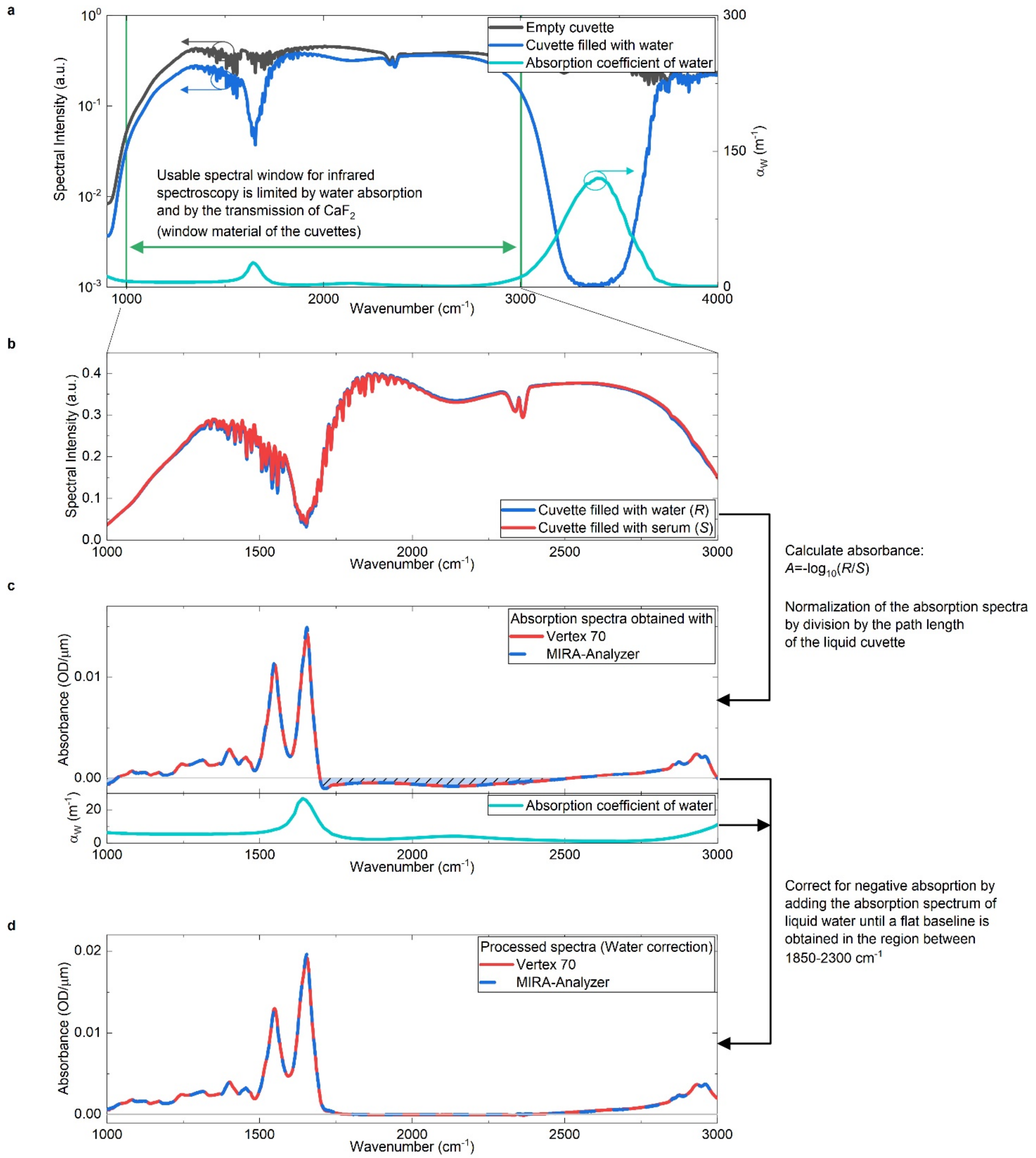

Supplementary Figure 1: *Acquisition and pre-processing of infrared spectra of liquid samples.* ***a****: Spectral intensity of the infrared light source after passing through the empty measurement cuvette (black line) and when it is filled with water (blue line). The strong absorption of liquid water (turquoise line) and the limited transmission of the window material (CaF_2 – not shown) limits the useable spectral window for spectroscopy (green double-arrow). Any incomplete filling or drying of the sample would be detectable by a strong change of the spectra in comparison to the water reference (blue line).* ***b****: Spectral intensity of the infrared light source when the cuvette is filled with water (blue line) and with blood serum (red line). Please note that the power spectra shown in* ***a*** *and* ***b*** *are for demonstration purposes only, and had to be performed with a different measurement cuvette (AquaSpec™, Bruker) and a different spectrometer (Vertex 70, Bruker) for technical reasons. This setup is similar to the experimental setup (MIRA-Analyzer, microbiolytics GmbH) used throughout the measurements presented in this work and provides qualitatively the same results (****c*** *and* ***d****).* ***c****: The resulting absorption spectra of quality control serum. Negative absorption is caused by the fact that the number of water molecules in the measurement volume is lower in the serum sample than in pure water, and therefore at some wavenumbers, less light is absorbed by the sample than in the reference. This measurement artefact can be compensated by adding a known water absorption spectrum[1] (****c*** *– turquoise line) until (****d****) a flat baseline in the range of 1850-2300 wavenumbers is reached.*

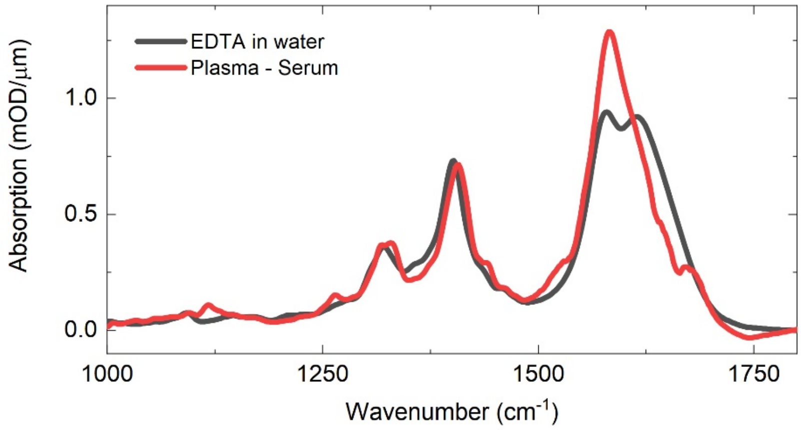

Supplementary Figure 2: *Difference between the average serum and plasma infrared (IR) spectrum compared to the absorption IR spectrum of EDTA-Ca2+ dissolved in water.*

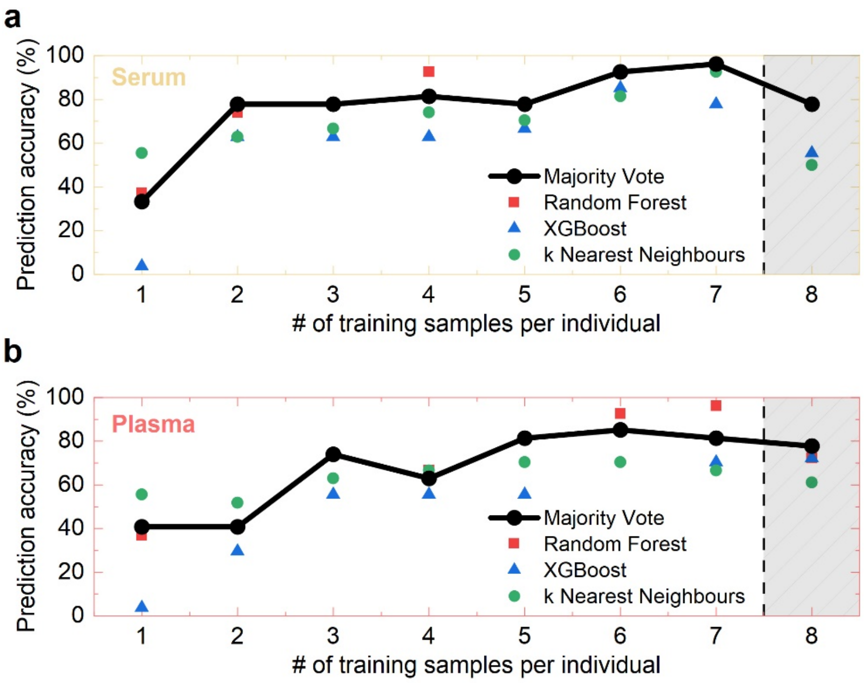

Supplementary Figure 3: *Learning curves of prediction models generated using different algorithms (random forests, gradient boosted trees (XGBoost) and k-nearest neighbours) based on infrared molecular fingerprints (IMFs) of* ***(a)*** *blood serum and* ***(b)*** *plasma. Note that the accuracy of person identification increases when data of more blood draws are included for training and drops to slightly below 80% (shaded area) when applied to IMFs sampled 6 months later, resembling the difference between the shorter- and longer-term biological variability. The selected classification models for these plots are obtained by tuning the algorithms' hyperparameters to the following sets of values:*

- *Random Forest: n_estimators=3160, others at default values of Scikit-Learn (v. 0.20.3) [see https://scikit-learn.org/stable/modules/generated/sklearn.ensemble.RandomForestClassifier.html for more information]*
- *XGBoost: n_estimators=100, others at default values of XGBoost (v. 0.80) [see https://xgboost.readthedocs.io/en/latest/parameter.html for more information]*
- *K-NN: n_neighbors=1, others at default values of Scikit-Learn (v. 0.20.3) [see https://scikit-learn.org/stable/modules/generated/sklearn.neighbors.KNeighborsClassifier.html#sklearn.neighbors.KNeighborsClassifier for more information]*

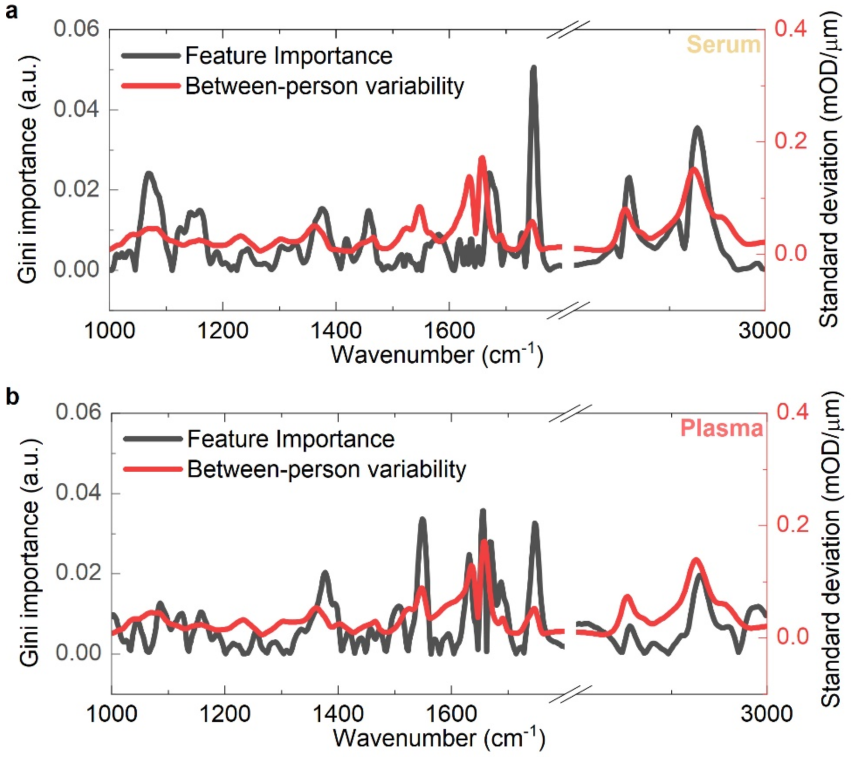

Supplementary Figure 4: *Gini importance (mean decrease in Gini impurity) of infrared spectral features (grey line) extracted by application of random forest algorithm used for Fig. 4d compared to the between-person variability (red line) of* ***(a)*** *serum and* ***(b)*** *plasma IMFs.*

Supplementary Table 3: *Gini importance (mean decrease in Gini impurity) of infrared intensity ratios – extracted by application of random forest algorithm – when the classification of individuals is based on intensity ratios instead of entire full infrared fingerprint spectra. Note that ratios with low Index of Individuality (*$\boldsymbol{II}$*) correlate with high Gini importance. Gini importance provides a means of measuring the relevance of each spectral feature with regard to the classification. It can be interpreted as a relative (and not absolute) measure of the importance of each feature and thus its values can be interpreted as high or low only relative to each other. Green-shaded cells correspond to larger numbers, while the red-shaded cells correspond to low numbers.*

	Serum			Plasma		
**Peak ratio:**	**Value ±** S_B^2	$\boldsymbol{II}$	**Gini Importance**	**Value ±** S_B^2	$\boldsymbol{II}$	**Gini Importance**
I_{1635}/I_{1654}	0.756 ± 0.012	0.231	0.086	0.771 ± 0.012	0.274	0.082
I_{1546}/I_{1655}	0.635 ± 0.003	0.613	0.034	0.638 ± 0.003	1.231	0.018
$I_{1655}/(I_{1655}+I_{1548})$	0.610 ± 0.001	0.625	0.033	0.608 ± 0.001	1.232	0.018
$I_{1684}/(I_{1655}+I_{1548})$	0.213 ± 0.002	0.481	0.038	0.218 ± 0.002	0.687	0.031
$I_{1515}/(I_{1655}+I_{1548})$	0.174 ± 0.002	0.268	0.071	0.175 ± 0.002	0.339	0.068
I_{2959}/I_{2931}	0.993 ± 0.020	0.521	0.025	1.005 ± 0.019	0.496	0.029
$(I_{2855}+I_{2927})/(I_{2962}+I_{2871})$	0.952 ± 0.023	0.547	0.026	0.942 ± 0.021	0.518	0.030
$(I_{2851}+I_{2927})/(I_{1655}+I_{1548})$	0.178 ± 0.007	0.484	0.026	0.179 ± 0.007	0.459	0.034
$I_{1239}/(I_{2851}+I_{2927})$	0.424 ± 0.013	0.542	0.025	0.422 ± 0.011	0.521	0.035
I_{1741}/I_{1640}	0.029 ± 0.003	0.592	0.030	0.029 ± 0.002	0.573	0.034
I_{1740}/I_{1400}	0.118 ± 0.012	0.589	0.027	0.107 ± 0.009	0.590	0.033
I_{2852}/I_{1400}	0.500 ± 0.018	0.539	0.025	0.454 ± 0.014	0.564	0.027
I_{1450}/I_{1539}	0.287 ± 0.003	0.301	0.059	0.297 ± 0.003	0.343	0.060
I_{1240}/I_{1517}	0.408 ± 0.004	0.512	0.028	0.407 ± 0.004	0.571	0.030
I_{1045}/I_{1545}	0.109 ± 0.003	0.405	0.042	0.109 ± 0.003	0.475	0.038
I_{1080}/I_{1550}	0.145 ± 0.004	0.397	0.047	0.143 ± 0.004	0.444	0.050
I_{1060}/I_{1230}	0.705 ± 0.013	0.430	0.052	0.697 ± 0.012	0.548	0.040
I_{1170}/I_{1080}	0.905 ± 0.017	0.504	0.044	0.918 ± 0.016	0.542	0.050
I_{1030}/I_{1080}	0.626 ± 0.006	0.806	0.044	0.636 ± 0.005	1.014	0.040
I_{1080}/I_{1243}	0.726 ± 0.014	0.457	0.045	0.717 ± 0.013	0.563	0.040
$I_{1587}/(I_{1655}+I_{1548})$	0.145 ± 0.002	0.452	0.046	0.178 ± 0.002	0.563	0.053
I_{1156}/I_{1171}	0.894 ± 0.007	0.450	0.053	0.898 ± 0.006	0.490	0.059
I_{1243}/I_{1314}	0.856 ± 0.013	0.410	0.050	0.802 ± 0.013	0.561	0.039
I_{1453}/I_{1400}	0.801 ± 0.006	0.434	0.043	0.737 ± 0.007	0.614	0.061

Supplementary Table 4: *Summary of the classification accuracy using different sets of infrared spectral features.*

	Train set (8-fold cross-validation)	Test Set
Plasma intensity ratios	75 %	56 %
Serum intensity ratios	85 %	61 %
Plasma (full spectra)	89 %	77 %
Serum (full spectra)	95 %	78 %

Supplementary Table 5: *Correlation matrix of intensity ratios taken from normalised infrared spectra of serum samples. Green-shaded cell correspond to larger numbers, while the red-shaded cells correspond to low numbers.*

	1635/1654	1546/1655	1655/(1655+1548)	1684/(1655+1548)	1515/(1655+1548)	2959/2931	(2855+2927)/(2962+2871)	(2851+2927)/(1655+1548)	1239/(2851+2927)	1741/1640	1740/1400	2852/1400	1450/1539	1240/1517	1045/1545	1080/1550	1060/1230	1170/1080	1030/1080	1080/1243	1587/(1655+1548)	1156/1171	1243/1314	1453/1400
**1635/1654**	1.00	0.43	-0.42	0.87	0.94	-0.36	0.35	0.44	-0.06	0.23	0.30	0.28	0.86	0.49	0.70	0.65	0.27	-0.51	0.05	0.27	0.85	0.80	0.75	0.766
**1546/1655**	0.43	1.00	-1.00	0.02	0.67	-0.04	0.04	0.10	0.09	0.02	0.04	0.01	0.19	-0.13	0.10	0.05	-0.04	-0.15	-0.17	-0.08	0.13	0.34	0.72	0.455
**1655/(1655+1548)**	-0.42	-1.00	1.00	-0.01	-0.66	0.06	-0.06	-0.12	-0.06	-0.04	-0.06	-0.04	-0.21	0.11	-0.12	-0.08	0.01	0.17	0.20	0.04	-0.13	-0.33	-0.74	-0.47
**1684/(1655+1548)**	0.87	0.02	-0.01	1.00	0.73	-0.26	0.25	0.32	0.01	0.17	0.23	0.18	0.76	0.48	0.63	0.57	0.21	-0.40	0.27	0.20	0.88	0.73	0.38	0.519
**1515/(1655+1548)**	0.94	0.67	-0.66	0.73	1.00	-0.25	0.24	0.33	0.04	0.14	0.20	0.17	0.74	0.29	0.60	0.53	0.21	-0.46	0.06	0.19	0.74	0.80	0.82	0.735
**2959/2931**	-0.36	-0.04	0.06	-0.26	-0.25	1.00	-1.00	-0.98	0.93	-0.96	-0.96	-0.98	-0.69	-0.88	-0.52	-0.62	-0.29	0.06	0.35	-0.40	-0.32	-0.26	-0.54	-0.71
**(2855+2927)/(2962+2871)**	0.35	0.04	-0.06	0.25	0.24	-1.00	1.00	0.98	-0.93	0.97	0.97	0.97	0.67	0.87	0.50	0.59	0.26	-0.03	-0.36	0.37	0.31	0.25	0.53	0.693
**(2851+2927)/(1655+1548)**	0.44	0.10	-0.12	0.32	0.33	-0.98	0.98	1.00	-0.92	0.94	0.95	0.98	0.74	0.89	0.57	0.67	0.33	-0.14	-0.39	0.43	0.38	0.27	0.59	0.754
**1239/(2851+2927)**	-0.06	0.09	-0.06	0.01	0.04	0.93	-0.93	-0.92	1.00	-0.93	-0.92	-0.97	-0.46	-0.75	-0.32	-0.45	-0.24	-0.07	0.45	-0.36	-0.08	0.05	-0.32	-0.51
**1741/1640**	0.23	0.02	-0.04	0.17	0.14	-0.96	0.97	0.94	-0.93	1.00	1.00	0.96	0.54	0.81	0.33	0.44	0.10	0.16	-0.39	0.22	0.18	0.10	0.46	0.621
**1740/1400**	0.30	0.04	-0.06	0.23	0.20	-0.96	0.97	0.95	-0.92	1.00	1.00	0.96	0.59	0.83	0.37	0.48	0.11	0.12	-0.39	0.23	0.24	0.15	0.50	0.669
**2852/1400**	0.28	0.01	-0.04	0.18	0.17	-0.98	0.97	0.98	-0.97	0.96	0.96	1.00	0.63	0.85	0.46	0.59	0.29	-0.05	-0.45	0.41	0.24	0.12	0.50	0.681
**1450/1539**	0.86	0.19	-0.21	0.76	0.74	-0.69	0.67	0.74	-0.46	0.54	0.59	0.63	1.00	0.78	0.83	0.83	0.46	-0.50	-0.05	0.50	0.83	0.69	0.71	0.866
**1240/1517**	0.49	-0.13	0.11	0.48	0.29	-0.88	0.87	0.89	-0.75	0.81	0.83	0.85	0.78	1.00	0.66	0.75	0.38	-0.27	-0.28	0.48	0.47	0.31	0.46	0.646
**1045/1545**	0.70	0.10	-0.12	0.63	0.60	-0.52	0.50	0.57	-0.32	0.33	0.37	0.46	0.83	0.66	1.00	0.96	0.83	-0.79	0.14	0.82	0.75	0.72	0.52	0.629
**1080/1550**	0.65	0.05	-0.08	0.57	0.53	-0.62	0.59	0.67	-0.45	0.44	0.48	0.59	0.83	0.75	0.96	1.00	0.84	-0.79	-0.10	0.88	0.66	0.61	0.54	0.653
**1060/1230**	0.27	-0.04	0.01	0.21	0.21	-0.29	0.26	0.33	-0.24	0.10	0.11	0.29	0.46	0.38	0.83	0.84	1.00	-0.86	0.03	0.98	0.38	0.40	0.22	0.279
**1170/1080**	-0.51	-0.15	0.17	-0.40	-0.46	0.06	-0.03	-0.14	-0.07	0.16	0.12	-0.05	-0.50	-0.27	-0.79	-0.79	-0.86	1.00	-0.01	-0.83	-0.50	-0.52	-0.32	-0.29
**1030/1080**	0.05	-0.17	0.20	0.27	0.06	0.35	-0.36	-0.39	0.45	-0.39	-0.39	-0.45	-0.05	-0.28	0.14	-0.10	0.03	-0.01	1.00	-0.11	0.25	0.37	-0.29	-0.25
**1080/1243**	0.27	-0.08	0.04	0.20	0.19	-0.40	0.37	0.43	-0.36	0.22	0.23	0.41	0.50	0.48	0.82	0.88	0.98	-0.83	-0.11	1.00	0.36	0.36	0.25	0.342
**1587/(1655+1548)**	0.85	0.13	-0.13	0.88	0.74	-0.32	0.31	0.38	-0.08	0.18	0.24	0.24	0.83	0.47	0.75	0.66	0.38	-0.50	0.25	0.36	1.00	0.77	0.46	0.604
**1156/1171**	0.80	0.34	-0.33	0.73	0.80	-0.26	0.25	0.27	0.05	0.10	0.15	0.12	0.69	0.31	0.72	0.61	0.40	-0.52	0.37	0.36	0.77	1.00	0.57	0.545
**1243/1314**	0.75	0.72	-0.74	0.38	0.82	-0.54	0.53	0.59	-0.32	0.46	0.50	0.50	0.71	0.46	0.52	0.54	0.22	-0.32	-0.29	0.25	0.46	0.57	1.00	0.904
**1453/1400**	0.77	0.46	-0.47	0.52	0.74	-0.71	0.69	0.75	-0.51	0.62	0.67	0.68	0.87	0.65	0.63	0.65	0.28	-0.29	-0.25	0.34	0.60	0.55	0.90	1

Supplementary Table 6: *Correlation matrix of intensity ratios taken from normalised infrared spectra of plasma samples. Green-shaded cell correspond to larger numbers, while the red-shaded cells correspond to low numbers.*

	1635/1654	1546/1655	1655/(1655+1548)	1684/(1655+1548)	1515/(1655+1548)	2959/2931	(2855+2927)/(2962+2871)	(2851+2927)/(1655+1548)	1239/(2851+2927)	1741/1640	1740/1400	2852/1400	1450/1539	1240/1517	1045/1545	1080/1550	1060/1230	1170/1080	1030/1080	1080/1243	1587/(1655+1548)	1156/1171	1243/1314	1453/1400
**1635/1654**	1.00	0.41	-0.42	0.79	0.93	-0.38	0.37	0.47	-0.05	0.25	0.32	0.27	0.85	0.46	0.66	0.63	0.25	-0.51	-0.01	0.25	0.60	0.81	0.69	0.522
**1546/1655**	0.41	1.00	-1.00	-0.15	0.62	-0.10	0.10	0.16	0.01	0.07	0.07	0.04	0.18	-0.19	0.06	0.07	0.04	-0.18	-0.29	0.03	0.03	0.37	0.69	0.362
**1655/(1655+1548)**	-0.42	-1.00	1.00	0.16	-0.62	0.13	-0.12	-0.18	0.02	-0.09	-0.09	-0.06	-0.20	0.17	-0.08	-0.09	-0.06	0.20	0.31	-0.06	-0.05	-0.37	-0.70	-0.359
**1684/(1655+1548)**	0.79	-0.15	0.16	1.00	0.65	-0.20	0.20	0.27	0.09	0.13	0.21	0.16	0.68	0.47	0.58	0.51	0.13	-0.36	0.28	0.11	0.53	0.63	0.26	0.355
**1515/(1655+1548)**	0.93	0.62	-0.62	0.65	1.00	-0.27	0.26	0.36	0.05	0.16	0.23	0.21	0.70	0.25	0.58	0.53	0.24	-0.51	-0.02	0.21	0.38	0.80	0.81	0.638
**2959/2931**	-0.38	-0.10	0.13	-0.20	-0.27	1.00	-1.00	-0.98	0.92	-0.96	-0.96	-0.94	-0.69	-0.84	-0.55	-0.63	-0.34	0.16	0.36	-0.45	-0.31	-0.31	-0.51	-0.416
**(2855+2927)/(2962+2871)**	0.37	0.10	-0.12	0.20	0.26	-1.00	1.00	0.98	-0.92	0.96	0.96	0.93	0.67	0.84	0.53	0.61	0.31	-0.13	-0.36	0.42	0.31	0.30	0.49	0.396
**(2851+2927)/(1655+1548)**	0.47	0.16	-0.18	0.27	0.36	-0.98	0.98	1.00	-0.90	0.94	0.94	0.94	0.75	0.85	0.59	0.68	0.37	-0.23	-0.38	0.47	0.35	0.33	0.56	0.465
**1239/(2851+2927)**	-0.05	0.01	0.02	0.09	0.05	0.92	-0.92	-0.90	1.00	-0.93	-0.89	-0.90	-0.45	-0.70	-0.32	-0.44	-0.28	-0.01	0.43	-0.39	-0.18	0.01	-0.26	-0.211
**1741/1640**	0.25	0.07	-0.09	0.13	0.16	-0.96	0.96	0.94	-0.93	1.00	0.99	0.93	0.55	0.79	0.38	0.47	0.18	0.05	-0.37	0.28	0.19	0.15	0.42	0.374
**1740/1400**	0.32	0.07	-0.09	0.21	0.23	-0.96	0.96	0.94	-0.89	0.99	1.00	0.95	0.59	0.82	0.43	0.52	0.20	0.00	-0.36	0.29	0.16	0.19	0.49	0.476
**2852/1400**	0.27	0.04	-0.06	0.16	0.21	-0.94	0.93	0.94	-0.90	0.93	0.95	1.00	0.57	0.82	0.51	0.61	0.36	-0.17	-0.40	0.45	0.05	0.14	0.53	0.58
**1450/1539**	0.85	0.18	-0.20	0.68	0.70	-0.69	0.67	0.75	-0.45	0.55	0.59	0.57	1.00	0.73	0.80	0.80	0.45	-0.52	-0.09	0.50	0.73	0.72	0.60	0.463
**1240/1517**	0.46	-0.19	0.17	0.47	0.25	-0.84	0.84	0.85	-0.70	0.79	0.82	0.82	0.73	1.00	0.65	0.72	0.37	-0.32	-0.25	0.45	0.38	0.29	0.37	0.373
**1045/1545**	0.66	0.06	-0.08	0.58	0.58	-0.55	0.53	0.59	-0.32	0.38	0.43	0.51	0.80	0.65	1.00	0.96	0.83	-0.82	0.13	0.81	0.49	0.72	0.54	0.522
**1080/1550**	0.63	0.07	-0.09	0.51	0.53	-0.63	0.61	0.68	-0.44	0.47	0.52	0.61	0.80	0.72	0.96	1.00	0.84	-0.83	-0.11	0.87	0.46	0.64	0.57	0.533
**1060/1230**	0.25	0.04	-0.06	0.13	0.24	-0.34	0.31	0.37	-0.28	0.18	0.20	0.36	0.45	0.37	0.83	0.84	1.00	-0.86	0.01	0.98	0.25	0.44	0.35	0.341
**1170/1080**	-0.51	-0.18	0.20	-0.36	-0.51	0.16	-0.13	-0.23	-0.01	0.05	0.00	-0.17	-0.52	-0.32	-0.82	-0.83	-0.86	1.00	0.04	-0.84	-0.32	-0.58	-0.45	-0.399
**1030/1080**	-0.01	-0.29	0.31	0.28	-0.02	0.36	-0.36	-0.38	0.43	-0.37	-0.36	-0.40	-0.09	-0.25	0.13	-0.11	0.01	0.04	1.00	-0.13	0.08	0.26	-0.30	-0.16
**1080/1243**	0.25	0.03	-0.06	0.11	0.21	-0.45	0.42	0.47	-0.39	0.28	0.29	0.45	0.50	0.45	0.81	0.87	0.98	-0.84	-0.13	1.00	0.30	0.42	0.35	0.318
**1587/(1655+1548)**	0.60	0.03	-0.05	0.53	0.38	-0.31	0.31	0.35	-0.18	0.19	0.16	0.05	0.73	0.38	0.49	0.46	0.25	-0.32	0.08	0.30	1.00	0.57	0.04	-0.206
**1156/1171**	0.81	0.37	-0.37	0.63	0.80	-0.31	0.30	0.33	0.01	0.15	0.19	0.14	0.72	0.29	0.72	0.64	0.44	-0.58	0.26	0.42	0.57	1.00	0.57	0.394
**1243/1314**	0.69	0.69	-0.70	0.26	0.81	-0.51	0.49	0.56	-0.26	0.42	0.49	0.53	0.60	0.37	0.54	0.57	0.35	-0.45	-0.30	0.35	0.04	0.57	1.00	0.849
**1453/1400**	0.52	0.36	-0.36	0.36	0.64	-0.42	0.40	0.47	-0.21	0.37	0.48	0.58	0.46	0.37	0.52	0.53	0.34	-0.40	-0.16	0.32	-0.21	0.39	0.85	1

Supplementary References

1. Segelstein, D. J. The complex refractive index of water. (1981).
