# Supplementary material for: Stability of person-specific blood-based infrared molecular fingerprints opens up prospects for health monitoring
Source: Nat Commun. 2021 Mar 8;12:1511. doi: 10.1038/s41467-021-21668-5 (PMC7940620; doi:10.1038/s41467-021-21668-5)
Supplement: Supplementary file 3 — Description of Additional Supplementary Files [file 41467_2021_21668_MOESM3_ESM.pdf]

Description of Additional Supplementary Files

File Name: Supplementary Data 1
Description: All unprocessed spectral data used in this study.
