# Supplementary material for: Stability of person-specific blood-based infrared molecular fingerprints opens up prospects for health monitoring
Source: Nat Commun. 2021 Mar 8;12:1511. doi: 10.1038/s41467-021-21668-5 (PMC7940620; doi:10.1038/s41467-021-21668-5)
Supplement: Supplementary file 5 — Reporting Summary [file 41467_2021_21668_MOESM5_ESM.pdf]

nature research

Corresponding author(s): Marinus Huber, Mihaela Zigman

Last updated by author(s): Feb 1, 2021

Reporting Summary

Nature Research wishes to improve the reproducibility of the work that we publish. This form provides structure for consistency and transparency in reporting. For further information on Nature Research policies, see our Editorial Policies and the Editorial Policy Checklist.

Statistics

For all statistical analyses, confirm that the following items are present in the figure legend, table legend, main text, or Methods section.

n/a	Confirmed	
☐	☒	The exact sample size (*n*) for each experimental group/condition, given as a discrete number and unit of measurement
☐	☒	A statement on whether measurements were taken from distinct samples or whether the same sample was measured repeatedly
☒	☐	The statistical test(s) used AND whether they are one- or two-sided<br>*Only common tests should be described solely by name; describe more complex techniques in the Methods section.*
☒	☐	A description of all covariates tested
☒	☐	A description of any assumptions or corrections, such as tests of normality and adjustment for multiple comparisons
☐	☒	A full description of the statistical parameters including central tendency (e.g. means) or other basic estimates (e.g. regression coefficient) AND variation (e.g. standard deviation) or associated estimates of uncertainty (e.g. confidence intervals)
☐	☒	For null hypothesis testing, the test statistic (e.g. *F*, *t*, *r*) with confidence intervals, effect sizes, degrees of freedom and *P* value noted<br>*Give P values as exact values whenever suitable.*
☒	☐	For Bayesian analysis, information on the choice of priors and Markov chain Monte Carlo settings
☐	☒	For hierarchical and complex designs, identification of the appropriate level for tests and full reporting of outcomes
☒	☐	Estimates of effect sizes (e.g. Cohen's *d*, Pearson's *r*), indicating how they were calculated

Our web collection on statistics for biologists contains articles on many of the points above.

Software and code

Policy information about availability of computer code

Data collection	No software was used.
Data analysis	Custom code was written using the Python (v.3.6.8) programing language and the open source machine-learning packages Scikit-Learn (v. 0.20.3) and XGBoost (v. 0.80). All steps of the data analysis are described in published literature, and the corresponding references can be found in the manuscript.<br>The custom code used for the production of the results presented in this manuscript is stored in a persistent repository at the Leibniz Supercomputing Center of the Bavarian Academy of Sciences and Humanities (LRZ), located in Garching, Germany. The code can be only shared upon reasonable request, as its correct use depends on the settings of the experimental setup and the measuring device and should therefore be clarified with the authors.

For manuscripts utilizing custom algorithms or software that are central to the research but not yet described in published literature, software must be made available to editors and reviewers. We strongly encourage code deposition in a community repository (e.g. GitHub). See the Nature Research guidelines for submitting code & software for further information.

Data

Policy information about availability of data

All manuscripts must include a data availability statement. This statement should provide the following information, where applicable:

- Accession codes, unique identifiers, or web links for publicly available datasets
- A list of figures that have associated raw data
- A description of any restrictions on data availability

The authors declare that the main data supporting the findings of this study are available within the article and its Supplementary Information files.

Field-specific reporting

Please select the one below that is the best fit for your research. If you are not sure, read the appropriate sections before making your selection.

☒ Life sciences ☐ Behavioural & social sciences ☐ Ecological, evolutionary & environmental sciences

For a reference copy of the document with all sections, see nature.com/documents/nr-reporting-summary-flat.pdf

Life sciences study design

All studies must disclose on these points even when the disclosure is negative.

Sample size	The required sample size was determined by a statistical power calculation prior to the study. A summarized version of the sample size calculation is included in the manuscript. The complete calculation is available on request from the corresponding author.
Data exclusions	Data from two individuals were completely excluded as they only participated in the sampling at the first or second sampling point. Therefore, no longitudinal analysis was performed for these two individuals, and these two volunteers were excluded from all analyses.
Replication	No replicates were measured. However, quality control serum samples were measured in all measurements to evaluate the measurement error. We found that the measurement error was very small compared to the biological variability of the samples (explained in the Results section). In addition, the results obtained from plasma and serum samples from the same donors yielded similar data, indicating that no technical variance or device variation affected the measurement results.
Randomization	All samples of each set (blood plasma as well as blood serum) were measured in a random order.
Blinding	Blinding was not relevant for our study, as neither a clinical trial nor a case-control study was conducted. The only form of blinding that was performed was the removal of the target labels during the testing of the classification models.

Reporting for specific materials, systems and methods

We require information from authors about some types of materials, experimental systems and methods used in many studies. Here, indicate whether each material, system or method listed is relevant to your study. If you are not sure if a list item applies to your research, read the appropriate section before selecting a response.

Materials & experimental systems

n/a	Involved in the study	
☒	☐	Antibodies
☒	☐	Eukaryotic cell lines
☒	☐	Palaeontology and archaeology
☒	☐	Animals and other organisms
☐	☒	Human research participants
☒	☐	Clinical data
☒	☐	Dual use research of concern

Methods

n/a	Involved in the study	
☒	☐	ChIP-seq
☒	☐	Flow cytometry
☒	☐	MRI-based neuroimaging

Human research participants

Policy information about studies involving human research participants

Population characteristics	Age of the participants ranged from 20 to 71 years with a mean of 39.6 years (±14.0 years, STD). 54.5% of the participants were female. This represents a typical cross-section of an adult population with some small bias towards younger age and the female gender.
Recruitment	Non-symptomatic, healthy volounteers were recruited at the local research site. The only selection criteria was that all participants did not have any symptoms nor severe or chronic diseases and were not undergoing any medical treatment. The aim was to recruit more than 30 volunteers (see also Calculation of sample size).<br>Possible biases were taken into account as far as possible. One limitation of the study is that the cohort of participants has specific demographic characteristics. However, no detailed hypothesis can be made that links demographic characteristics to the derived results. We expect our results and methodology to be generalisable.
Ethics oversight	The study was reviewed and approved by the Ethikkommission bei der LMU München (EK 20170820) (Ref. Nr. 17-532).

Note that full information on the approval of the study protocol must also be provided in the manuscript.
